# Reconstructing the lifelong history of cells and tissues via somatic mutation analysis

**DOI:** 10.1007/s00018-025-05946-9

**Published:** 2025-12-08

**Authors:** Sipontina Faienza, Jean Piero Margaria, Irene Franco

**Affiliations:** 1 Università Vita-Salute San Raffaele, Milan, 20132 Italy; 2 Somatic Mutation Mechanisms Unit, Division of Genetics and Cell Biology, IRCCS Ospedale San Raffaele, Milan, 20132 Italy

**Keywords:** Somatic mutation, Aging, Genome, Single cell

## Abstract

During a lifetime, normal cells accumulate thousands of changes in their genome sequence. These changes, termed somatic mutations, have mostly been studied in the context of cancer, but their presence in normal tissues is ubiquitous and widespread. Somatic mutation accompanies the aging process and is influenced by genetic and environmental factors. Differently from gene expression or imaging data, which fluctuate over time, somatic variants are non-reversible marks in the genome and accumulate over time. This property can be exploited to track the history of a cell, from conception to old age, providing information that cannot be acquired via classical histological tissue inspection nor other types of omics data. Mutations can track embryonic development, measure how clones compete in a tissue over time, or report the mutational processes active in cells and tissues throughout life. We discuss selected examples and emphasize how somatic mutation analysis can enable expanding applications at the service of physiology and cell biology, as well as a deeper understanding of the aging process.

## Introduction

Aging research extensively uses cellular and animal models to discover and validate cause-effect relationships at cellular and organismal levels. These models have the advantage of simplifying the study of a complex phenomenon, but can be significantly distant from events actually occurring in the human body under physiological or pathological conditions. Cell-based assays lack the complexity of interactions among different cell types and systems in the body. Animal models provide this complexity but introduce species-specific differences. The environmental stimuli are another important confounder, as human-specific activities and exposures are often hard to reproduce in animals in captivity. Moreover, the lifespan in humans and the most widely used animal models, i.e. small rodents, is dramatically different. For these reasons, it is crucial to implement methods to discover and validate the events that govern age-related physiology and pathology directly in human tissue samples.

More affordable and advanced sequencing technologies have recently become an ideal tool to extract information from human samples. Among other innovations, genomics has powered single-cell research, deepening our understanding of complex tissues and their dynamic changes over time [1]. Although single-cell analyses predominantly focus on exploring the transcriptome and epigenome, the genome sequence of individual cells is also emerging as a relevant source of information about tissue and cell biology. The genome sequence is classically described as a stable entity that should be maintained unaltered during a lifetime. Instead, sequencing studies in the last years have shown that every cell in the body accumulates mutations with time [2–4]. These alterations are referred to as somatic variants or somatic mutations.

Somatic genetic changes are a completely normal and physiological phenomenon generated by the continuous activity of exogenous and endogenous mutation processes [5]. The cellular system deputed to genome maintenance is extraordinarily efficient. Nonetheless, a tiny fraction of the billions bases of the genome sequence contained in each cell are altered over time [6]. Large insertions and deletions are not found in every cell, hence difficult to quantify. Conversely, studies have precisely quantified the average accumulation of small variants in various normal tissues. In particular, new single nucleotide variants (SNVs) and small insertions/deletions (indels) occur yearly in each cell of the body, at a rate that changes across tissues and that ranges between 10–80 SNVs and 1–10 indels per genome[2, 7]. Importantly, somatic mutations are not reversible. These changes are passed on to the next generation of cells, which keep accumulating mutations over the years. The result of this process is that every cell in a tissue has a slightly different genome compared to the surrounding cells.

**Fig. 1:**
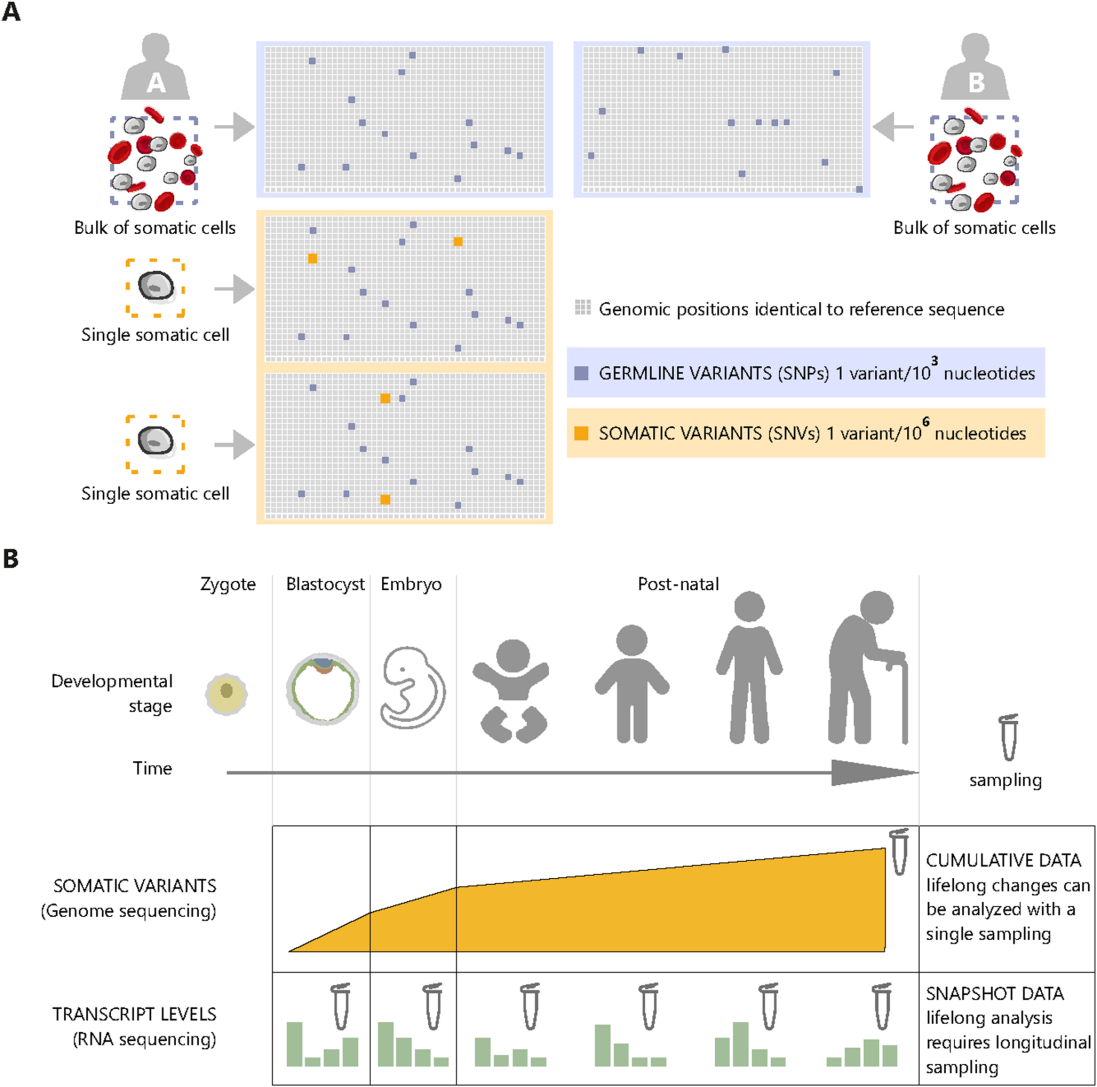
Lifelong accumulation of somatic variants. **A** Frequency of germline and somatic variants in the genome sequence. Top: Representation of germline variants (germline single nucleotide polymorphisms, SNPs) in a section of the genome sequence of two distinct individuals (A and B), compared to the reference sequence. Bottom: Representation of a portion of the genome sequence in two different cells of the same individual and the distribution of somatic single nucleotide variants (SNVs, orange, different in each cell) and germline SNPs (blue, same in both cells). **B** Accumulation of somatic variants during life and the unique perspective offered by somatic mutation data. The figure illustrates the developmental trajectory of the human organism and the accumulation of somatic variants in cells and tissues from the stage of zygote to postnatal age. Since genetic changes are non-reversible, a tissue sample contains all the genetic alterations that have accumulated in its cells and their lineages throughout an individual's life. Thanks to this property, somatic mutation data obtained from human tissue samples can inform on lifelong events, such as developmental and lineage history of individual cells, as well as mutagen exposure at any time during life. Unlike somatic variants, gene expression levels (as well as other types of data that can be captured by OMICS technologies, such as proteomics and metabolomics) fluctuate over time. Therefore, these types of data only offer a transient snapshot of the tissue at a given time point

**Fig. 2:**
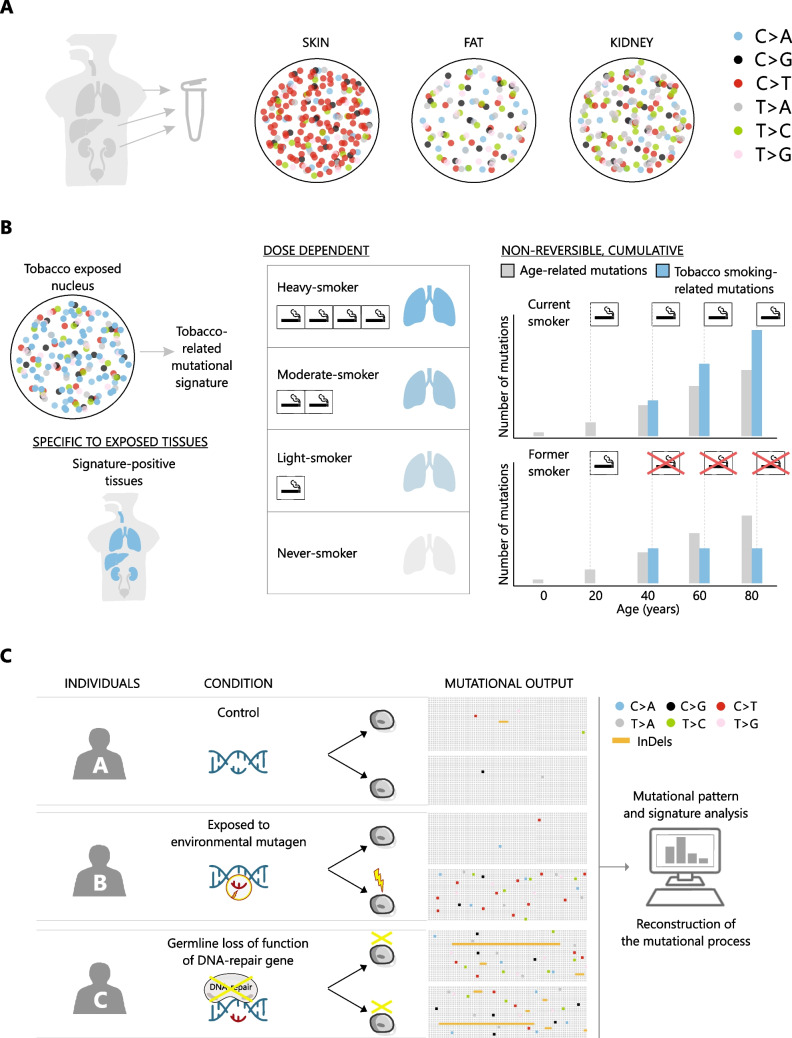
Mutational patterns as a report of mutation processes occurring during life. **A** Schematic visualization of mutation spectra in different cells. Somatic single nucleotide variants are represented as dots in the nucleus of cells derived from different tissues of the same individual. A specific color is assigned to each type of base substitution. This representation of somatic mutation spectra in different cells is an example of a simple color-based pattern that is derived from exposure to tissue-specific mutagens, i.e. sun exposure causing substitutions of a C with a T (C > T). **B** General characteristics of mutational signatures exemplified by exposure to a common environmental mutagen (tobacco). Mutation spectra can be divided in single components (mutational signatures), each one describing the mutational output of a specific mutation process (top left). The signal of a specific mutational signature is found only in tissues where the mutational process is active (“specific to exposed tissues”, bottom left), correlates with the activity levels of the mutational process (“dose dependent”, middle) and is not reversible, therefore detected even if the process has ceased many years before sample collection (“non-reversible, cumulative”, right). **C** Study of mutational patterns and signatures in cells and tissues can inform on exposure and activity of environmental mutagens and provide an accurate description of the molecular activity of DNA-repair enzymes and complexes

Compared to germline variants, somatic variants are quantitatively minor (Fig. 1A). Two cells from 2 individuals generally differ by 1 base every 10^3^ bases, while two distinct cells within an individual differ by 1 base every 10^6^–10^7^ bases [4]. Germline variance determines easy-to-appreciate differences among individuals. Conversely, somatic variance is relatively under-investigated and its functional impact is difficult to evaluate [8]. Nonetheless, when correctly detected and interpreted, somatic variants can be a unique tool to answer complex questions, including how tissues age (Fig. 1B). For example, cells might acquire mutations that modify their activity during life. Researchers can track these events in time and study subsequent cellular perturbations [9–11]. In addition, instead of single events, mutations in a genome can be analyzed as aggregated data that produce patterns and signatures [12] (Fig. 2A-B). Similar to other types of cell biology data (e.g. cell morphology, accumulation of a specific protein in a given subcellular compartment, proliferation rate in culture, etc.), these patterns can be quantified and analyzed. For example, they can be compared in different populations (e.g. exposed vs not exposed to a specific stimulus, mutated vs wild-type for a given gene) and used to study the molecular mechanism underlying mutation (Fig. 2C).

This review explains the rationale for using somatic mutation data to acquire information that cannot be acquired with classical analyses, nor from other types of omics data. Furthermore, we report selected examples illustrating how the analysis of somatic variants in human samples has been exploited to understand complex processes occurring in human tissues during a lifetime.

## Somatic variants as natural barcodes to track the cell’s history

Similar to fluorescent proteins used in lineage tracing studies in animal models, genetic changes identified by genome sequencing can be used to trace a single cell and its progeny in the human body. This principle has been applied for longitudinal tracking of hematopoietic stem cells in patients undergoing gene therapy [13]. In these cases, viral vectors able to integrate in the genome are used for ex vivo delivery of therapeutic genetic material into hematopoietic stem and progenitor cells, which are then re-infused into the patient. The integration site of the viral vector is unique in each cell and constitutes an attractive and permanent mark that can be easily retrieved by targeted sequencing of the blood of the patient. Mutation data are then used to recognize and measure the progeny of each stem cell, months and years after transplant [13]. Similar to these artificial mutations, spontaneously occurring somatic variants can be used for barcoding single cells. Moreover, multiple variants can be traced at once, providing many advantages that will be covered in the next paragraphs.

Somatic variants accumulate throughout life and remain permanently embedded in the genome. Genetic changes are transferred to descendant cells, which in turn accumulate additional variants in a tissue-specific manner [14]. A notable difference between germline and somatic variants is that the former are shared by all cells in the body, while the latter are only found in a fraction of cells. This happens because somatic changes are post-zygotic, i.e. they occur after the first embryonic division and are not propagated to all cells [4]. The degree of propagation of somatic variants in tissues is termed mosaicism and is an important property that has been exploited in several applications. The mosaicism of somatic alterations in a sample is quantified by the variant allele frequency (VAF). High VAF for one particular variant means that most cells in the sample share that variant (e.g. VAF = 0.5, all cells in the sample carry that variant in heterozygosity). Low VAF, instead, indicates that a small percentage of cells share the variant, while the other cells in the sample are derived from different somatic lineages [15].

Variants with higher and lower VAFs are commonly identified when sequencing a piece of tissue, i.e. a population of somatic cells. Because identical variants are unlikely to arise independently in two different cells, shared mutations denote a common ancestor. High-VAF variants in a tissue sample can be variants with no functional consequences for the cell, but generated by somatic events that occurred early during development. In this case, the passenger variant is transmitted to a high fraction of cells of the organism and is likely to be observed in multiple tissues across the body [16, 17]. Alternatively, high VAF can be due to local expansion of a single cell that carries a mutation that confers selective advantage. In this case, the variant is only detectable in a specific tissue or tissue portion and does not necessarily occur during early development. Rather the opposite, expansion of local clones becomes more common as tissues age or in tissues experiencing specific conditions imposing a selective pressure [18–20].

Hereafter, we present examples of how SNVs have been used as permanent molecular barcodes, providing insights into embryonic development and the clonal composition of human tissues.

### Somatic variants as a tool for reconstructing embryonic development

Single or unique combinations of SNVs constitute an attractive natural feature to label individual cells in humans. This analysis can reconstruct the complexity of organogenesis and the branching of cell lineages that start from the zygote and lead to mature tissues. SNVs that are shared among different cells are used to reconstruct clades, i.e. groups that include a common ancestor and its descendants. Distinct SNVs are used to define distinct clades and each branch of a lineage tree. When enough data are available, it is possible to generate a complete phylogenetic reconstruction, tracing every cell back to the initial two cells that gave rise to the entire organism [14, 15].

SNVs were first exploited for lineage tracing in mice. Researchers reconstructed the early cell division and developmental lineage tree by sequencing single genomes of adult tissues originating from distinct embryonic layers (stomach gland, small intestine, and colonic crypt) [21]. This study demonstrated that the first cells of the embryo contribute asymmetrically to adult tissue formation and that each organ develops from the cooperation of several distinct embryonic progenitors [21]. Subsequent work reconstructed phylogenetic trees using whole-genome sequencing (WGS) of single cells from various human tissues [15, 16, 22, 23]. These analyses confirmed that the contribution to the phylogenies of the two initial cells of the embryo is uneven also in humans [15, 23]. In the brain, 22% of somatic mutations shared by at least 10% of neurons were also detected in heart, spleen, and liver, and more than half were detected in at least two non-brain tissues, indicating that these variants were likely acquired before gastrulation. Moreover, the analysis of patterns of clonal distribution in non-brain organs revealed the asymmetric contribution to tissues even at later steps of gastrulation and organogenesis [16]. Finally, using SNV data, researchers could infer that cell specification and localization to the final tissue is established as early as the 9th-17th cell division of the embryo [23] and could quantitatively define key processes governing the development of human brain [17, 24, 25].

Examples reported so far illustrate the unicity of somatic mutation data over other omics approaches. Transcriptomics, proteomics, and metabolomics data offer a snapshot of cellular states at the specific time of sampling. Conversely, somatic mutations are cumulative records of cellular events occurred during the entire lifetime (Fig. 1B).

### Somatic variants can assess the clonal composition of tissues: examples from the immune system and the aging blood

Somatic mutation analysis is an ideal method to measure the clonal composition of a tissue, i.e. the phylogenetic relationships among cells composing the tissue and the possible presence of populations of somatic cells that have locally expanded from a single ancestor. Using somatic mutations, the size of each clone and the timing of its expansion can be determined [19]. Somatic mutations are not only used as markers of clonal expansion. In fact, they can also be “drivers” of clonal expansion. Most somatic variants are neutral and do not affect cell fitness. But occasionally, a somatic mutation provides a proliferative advantage to the cell in which it occurs and favors its relative expansion compared to other cells and clones in the tissue. This phenomenon is defined as positive selection. Clonal expansion driven by selection ubiquitously occurs in adult tissues, particularly the blood, and impacts physiological processes as well as age-related phenotypes [19, 26].

Clonal expansion dynamics are a fundamental principle governing the physiology of the immune system. Positive selection and expansion of B cell clones is the strategy adopted by the body to strengthen its immune defense against pathogens. The immune system utilizes somatic mutations to generate cellular diversity and produce antibodies and receptors with affinity for the wide variety of antigens that may be encountered during life. Antibody diversification begins in immature B lymphocytes through V(D)J recombination, which rearranges gene segments to form the antigen-binding region of immunoglobulins. While this process creates a diverse primary antibody repertoire, it does not produce high-affinity antibodies [27]. Upon antigen exposure, B cells undergo somatic hypermutation (SHM) to increase antigen-binding affinities in the germinal centers [28]. SHM primarily introduces point mutations, but also insertions and deletions within the variable region of immunoglobulin genes [29]. The SHM is followed by affinity-based selection in B-cells, wherein only clones with the highest affinity for the antigen are positively selected to proliferate and differentiate into memory B cells or antibody-secreting cells [18].

Interestingly, WGS of normal B cells has revealed off-target mutations associated with SHM [30]. The somatic mutation burden increases progressively along with lymphocyte differentiation, with memory B and T cells accumulating hundreds to thousands more mutations than their naïve counterparts, which in turn show a higher burden than hematopoietic stem cells [31]. Notably, approximately 11% of non-synonymous off-target somatic variants confer a selective advantage to lymphocytes, suggesting that positive selection also shapes the clonal dynamics of normal immune cells [31, 32].

The entire hematopoietic system is sustained by the continuous and dynamic expansion of pools of cells originating from blood-forming progenitors. Lineage trees inferred from somatic SNVs detected in single blood progenitors can establish the clonal dynamics within the hematopoietic compartment of an individual [33]. Interestingly, the clonal composition of the hematopoietic system changes as we age. The blood compartment was found to be highly polyclonal in younger individuals, where every clone generated less than 1% of blood-forming progenitors. By contrast, adults older than 70 experienced a loss of clonal diversity, with the appearance of clones that expanded more than others and generated up to 30% of blood-forming progenitors. This pattern was observed in all 70 + individuals analyzed in the study, suggesting that clonal diversity loss in the hematopoietic system is a normal aging process [10].

Intensive research is now trying to establish what genes confer selective advantage to hematopoietic stem cells and their progeny. Different approaches confirmed that the lists of positively selected genes in normal clonal hematopoiesis and blood cancers only partially overlap. Moreover, mutated clones generally appear in the first decades of life and expand very slowly [10, 34, 35]. Expansion of mutated clones can also induce pathological consequences that disrupt the activity of the immune system. One example is given by mutations in the *PIGA* gene, which confer a growth advantage to hematopoietic stem cells, but multiple defects in differentiated compartments. These defects result in a complex immune-hematological condition, characterized by haemolysis, thrombosis and bone marrow failure, termed paroxysmal nocturnal hemoglobinuria [36, 37]. Instead, dominant clones that acquire somatic mutations in the *UBA1* gene expand and create a pro-inflammatory state across all differentiated lineages, poisoning normal hematopoiesis and causing a fast-progressing, autoinflammatory syndrome, termed VEXAS [38].

Somatic variants have also been used as lineage markers to reconstruct the phylogeny of the hematopoietic system after allogeneic hematopoietic cell transplantation [39]. A particularly significant question is how many transplanted cells can maintain blood production and contribute to the formation of mature blood cells. By comparing the clonal dynamics of recipients to the native clonal dynamics of donors, researchers quantified the lasting impact of hematopoietic cell transplantation on blood production. The study analyzed WGS data from 10 donor-recipient sibling pairs, spanning a period of 9 to 31 years after transplantation. Researchers evaluated how transplanted cells contribute to hematopoiesis and found a higher number of contributing cells when transplanted cells were derived from younger rather than older donors [39]. They also observed an accelerated decline in clonal diversity within recipients, possibly resembling an accelerated aging of the hematopoietic system. Loss of clonal diversity corresponded to 12 extra years of physiological aging in the recipient compared to the donor sibling [39].

### Somatic variants can assess clonal dynamics and the tissue response to pathological stimuli

In solid organs, tissue organization is determined by physical constraints and clonal dynamics are less prominent compared to the hematopoietic compartment. Nonetheless, the number and size of detectable clones have been found to generally increase with age and the dynamics of this phenomenon appear tissue-specific [40]. In some tissues, like the esophagus epithelium, aging is accompanied by extensive clonal expansion, and detectable clones occupy the majority of the epithelial layer after the age of 50 [41]. Conversely, other tissues, like the skeletal muscle, maintain clonal diversity and remain highly polyclonal until old age [42].

In normal tissues, clonal expansion is mostly interpreted as the first step to malignant transformation. In support, conditions that increase cancer risk (i.e. advanced age, alcohol and cigarette consumption) usually coincide with a higher number and size of detectable clones in human tissue samples [41, 43, 44]. Instead, the study of liver tissue evolution in chronic liver disease highlighted clonal expansion as a mechanism to cope with tissue stress [45, 46]. Chronic liver disease is induced by infections, alcohol consumption, and other forms of liver damage, which culminate in liver failure [47]. The chronic state of injury-repair promotes the selection of clones carrying somatic variants that confer a growth advantage. At the same time, epidemiologic data show a clear correlation between chronic liver diseases and the risk of developing liver cancer [48]. Surprisingly, an unbiased analysis of recurrently mutated genes in non-malignant liver samples from chronic liver disease patients showed that the most frequent, positively selected mutations are not located in cancer-driver genes, but in genes that modulate hepatocyte metabolism [45].

The most frequently mutated gene in cirrhotic human liver is *PKD1* [45, 46]. Polycystin-1, the product of *PKD1,* is mostly studied in the context of autosomal dominant polycystic kidney disease (ADPKD), a hereditary genetic condition characterized by the growth of kidney cysts that replace the normal tissue, eventually leading to kidney failure [49]. Loss of PKD1 induces significant changes in cellular metabolism [50]. Moreover, germline mutations in *PKD1* are known to cause bile-duct cysts in the liver [49]. However, the prominent role of *PKD1* mutations in clonal expansion of hepatocytes is unexpected. Mouse models of *Pkd1* loss in hepatocytes have shown a functional role of this gene in clonal expansion and tissue regeneration, specifically under liver-damaging conditions. *Pkd1* loss promoted adaptation to the stressing conditions, probably via a modulation of cellular metabolism. Remarkably, *Pkd1* loss in as few as 5% of mouse hepatocytes was enough to confer the beneficial effect, demonstrating that mutations with a low degree of mosaicism can have an important impact at whole tissue level [46].

To systemically measure how mosaic mutations impact liver regeneration, the same authors developed a platform called MOSAICS. This platform utilizes the CRISPR technology to generate a mosaic of somatically mutated clones in mouse liver and study the in vivo fitness under control or stress conditions [51]. In this system, the liver of Cas9 mice injected with a library of mutated guides is monitored by sequencing before and after a liver-damaging dietary treatment. During this time, mutations that confer selective advantage become over-represented in the tissue, and the clonal dynamics are precisely measured by the VAF of each guide. One important result of this first screening is that clonal selection was evident only in stress conditions, while clonal dynamics in control diet were too slow for detection. Moreover, the screening pointed to reduced lipid accumulation as a general strategy to gain clonal fitness in a damaged liver [51].

In the brain, instead, somatic mutations altering the PI3K (phosphoinositide 3-kinase)-mTOR (mammalian target of rapamycin) pathway have been recognized as drivers of malformation, particularly in focal cortical dysplasia (FCD) [52]. These mutations occur during cortical development and are found at variable levels of mosaicism. However, how PI3K-mTOR pathway mutations affect bearing cells and lead to disease pathophysiology is not clear. An innovative approach based on the detection of the specific somatic variant of interest, together with the acquisition of transcriptomic data in single cells, has enhanced our understanding of the disease mechanisms [9]. The method (termed “Genotyping Of Transcriptomes Enhanced with Nanopore sequencing” or GO-TEN), is based on a previously developed technology [53] and achieves high confidence genotyping of samples destined to single cell RNAseq. By analyzing the distribution of somatic mutations across cell types, researchers found that pathogenic variants do not generate new cell identities but rather are predominantly found in a specific subset of neuronal cells, possibly indicating cell-type specific positive selection when the pathogenic PI3K/mTOR variant is present [9].

Here, we have illustrated how somatic mutations can deepen our understanding of clonal dynamics in human tissues. Positively selected clones are over-represented in specific contexts and may exhibit beneficial effects helping the tissue adapt to pathological conditions. On the other hand, a reduction of clonal diversity is an important aspect of aging, and it certainly contributes to loss of tissue function and cancer.

## From single mutations to mutational patterns and signatures

In the last decade, the volume of somatic mutation data available for research has greatly increased, allowing unprecedented aggregated analyses of somatic mutations. In this type of study, the single mutations are not important, while thousands of mutations can generate meaningful patterns captured by statistical analyses (Fig. 2).

Some patterns are so evident that can be immediately spotted by simple visualizations (Fig. 2A). For example, a study collected whole-genome somatic mutation data from normal cells from different organs of an individual aged 69. For each cell, somatic mutations were represented as dots in the genome and each type of single base substitution (C > A; C > G; C > T; T > A; T > C; T > G) was given a specific color. While the plot representing kidney and fat cells showed a mixture of colors, the skin cell genomes were largely dominated by red-colored dots, corresponding to C > T transitions, suggestive of a specific mutation process [54]. The easy-to-guess mutagen is UV light, to which skin cells are exposed for decades. Chemical interactions of UV light with the DNA result in C to T transitions, which accumulate in each cell genome and constitute the predominant type of mutations in adult skin cells [55].

As different chemical reactions cause distinct DNA modifications, the spectrum of mutations detected in a genome can be used to reconstruct mutational processes that have occurred in the cell and its lineage during a lifetime [12]. Mutations are not only determined by the presence of endogenous and exogenous chemical agents able to interact with the DNA molecules (mutagens), but also by the activity of the endogenous machinery deputed to genome maintenance. In normal cells, the DNA is continuously exposed to the activity of endogenous and exogenous mutagens. However, cells can usually either repair the vast majority of these damages or eliminate the cells that are severely damaged [56]. For these reasons, normal cells accumulate somatic mutations at low pace. Perturbations of the steady state, including extraordinary exposure to mutagens or genetic and non-genetic impairment of the DNA repair machinery, can impress detectable marks in the DNA by increasing the frequency of specific types of mutations [5]. Every mutation process is expected to leave a specific pattern or “signature” of mutations. Given that each genome is exposed to multiple different mutagenic processes over a lifetime, the catalogue of somatic mutations in each genome consists of a mixture of mutational signatures. These different components can be separated by mathematical procedures. Moreover, for each genome is possible to determine what fraction of somatic mutations is attributable to each signature [12, 57, 58].

Here, we show how mutational signature analysis has been instrumental in understanding the exposure to mutagens and other cellular processes culminating in DNA changes in human samples (Fig. 2B and C). These concepts were initially established in the context of cancer, but now extend to cellular and tissue physiology.

### Mutational signatures can track mutagen exposure

The classification of SNVs based on 96 classes has been the most widely used method to analyze somatic mutation spectra. Mutational signatures derived from these spectra are constantly updated at the Catalogue of Somatic Mutations in Cancer (COSMIC) website and reported with progressive numbers, e.g. SBS (single base substitution) 1, SBS4, SBS7a, etc. [57].

A well-characterized SBS mutational signature is the SBS4, generated by exposure to tobacco in the tissues of smokers [59–61]. The mutation spectrum in the lung tissue of smokers is generated by chemical reactions between the components of tobacco (mutagens) and the DNA. Subsequent intervention of DNA repair is not necessarily able to revert DNA lesions caused by tobacco into the correct bases. The combined effect of these reactions produces a set of specific types of mutations that are reproducibly recognized as a mutational signature [12]. The signature associated with tobacco is paradigmatic and presents the following characteristics that can be generalized to all mutational signatures (Fig. 2B): it is found only in those tissues that are directly exposed to the mutagen [59]; it accumulates in a “dose-dependent” manner (in this case, it depends on the number of smoked cigarettes per year) [60]; it is cumulative throughout a lifetime and remains impressed in the genomes of individuals that abandoned smoking many years before sample biopsy [61].

Other examples of mutagen exposure are offered by chemotherapeutic drugs. Cancer patients are often treated with mutagenic drugs, which are purposely administrated for their ability to interact with the DNA and kill fast-proliferating cancer cells. In agreement with the mutagenic activity of these drugs, the pattern of somatic mutations detected in tumors that relapsed after chemotherapy was shaped by these treatments. Analysis of mutational signatures in cancer genomes could recognize the particular chemotherapeutic agent used by each patient and discover new therapy-related signals [62]. Mutational signature analyses have allowed to trace the mutagenic activity of chemotherapeutics also in non-cancer cells, such as normal blood, colon, and liver stem cells from cancer survivors [63, 64]. Mutations induced by a widely used chemotherapeutic drug (cisplatin) were also detected in de novo variants in children born from fathers who underwent chemotherapy, indicating that cisplatin treatment induces mutations in the male germline [65]. Follow up studies measuring the off-target activity of chemotherapy are critically important, as excessive mutation in normal cells can result in higher cancer risk. In agreement, a study traced the signature of cisplatin in secondary malignancies in cancer survivors, determining that liver and aesophagus are the tissues at highest risk of developing malignancies secondary to cisplatin treatment [66]. This information is clinically useful and helps refining strategies for active surveillance in cancer survivors. Another example of clinically relevant data provided by mutational signature analysis came from the in vitro evaluation of the mutational activity of a new chemotherapeutic drug, named CX-5461 [67]. The extraordinary capacity of this compound to induce mutations in human cells, superior to cisplatin, raised safety concerns for the progress of the clinical trial and stressed the need for an evaluation of the mutagenicity of any new compound before approval for use in human trials [67, 68].

Given the early stage of our knowledge on mutagens, it is plausible that unknown and never suspected DNA-interacting agents are present in everyday environments, at least in some parts of the world. This would possibly translate into an increased incidence of cancer. An international consortium involving researchers of the World Health Organization has launched the “Mutograph project”, a massive effort to collect cancer samples from different regions of the world [69]. Attention is placed on collecting samples from areas at high and low cancer incidence and correlating mutational signature data with epidemiologic and clinical data [69]. Esophageal cancers showed no geographic patterning of mutational signatures, despite regional differences in cancer incidence [70]. Conversely, the analysis of kidney cancers has identified some signatures that are restricted to specific areas of the world [71]. In the case of SBS22, the causative agent is known. This signature is induced by exposure to Aristolochic Acid, a carcinogen found in weeds that are either involuntarily ingested or used for traditional medicine [72]. In the case of the SBS12 mutational signature, instead, the specific enrichment in a specific Country (Japan) could not be explained [71]. Finally, this analysis led to the discovery of a kidney cancer-specific signature known as SBS40b. This signature has been found almost everywhere in the world, but the numbers of mutations generated by SBS40b were higher in Countries that registered a higher risk of developing kidney cancer, suggesting that exposure(s) leading to SBS40b might be a common stimulus favoring the development of kidney cancer [71].

More analyses exploring regional variation in cancers derived from the digestive/excretory tract will likely provide insights into mutagens introduced through the diet. Among the dietary factors suspected to facilitate cancer, a red meat-rich diet has raised a long-lasting interest [73]. A study has illustrated a mechanism by which red meat may facilitate tumorigenesis by increasing somatic mutation within the colonic epithelium [74]. In fact, meat-reach diets are associated with the occurrence of a specific mutational signature that induces changes of a C into a T. Key residues in the common colon oncogenes KRAS (Kirsten rat sarcoma virus) and PI3K are encoded by C bases. When these residues are mutated into T, they produce proteins with abnormal activity. In agreement with the known oncogenic activity of these mutated proteins, the meat-associated mutational signature and the oncogenic mutations are found to co-exist in cancer genomes more often than expected by chance [74].

In summary, somatic mutation studies have shown that our current knowledge about compounds or exposures causing mutations is incomplete. Mutational signatures are a precious tool for systematic analyses to test and uncover agents that threaten our tissues.

### Mutational patterns can track microbial infections

Among the agents that threaten genome integrity, there are microbial infections. Viral infections, including Epstein-Barr and papilloma viruses, have been known for decades for their ability to interact with the genome and produce cancer-causing genetic changes, like chromosomal alterations that activate oncogenes [75]. Bacterial infections are also emerging as an important life event that brings a burst of somatic mutations, most precisely single nucleotide variants. One example is a mutational signature discovered in the human intestinal epithelium, demonstrated to derive from the exposure to the toxin colibactin released by certain *E. Coli* strains (*pks*^+^ *E. coli)* [76, 77]. The new signature SBS88 was first identified in normal intestinal crypts of a subset of individuals [76]. The demonstration that the causative agent was colibactin came from an in vitro experiment where clonal human intestinal organoids were injected with *pks*^+^ *E. coli* [77]. A follow-up study characterized widespread mutagenic activity among *E. coli* strains and provided a better method to detect the colibactin signature in human samples. This method highlighted the signature in 12% of colon cancers. The infection has been traced to the first decades of life. Cancer patients exhibiting the colibactin signature showed earlier cancer incidence compared to colibactin-signature negative tumor bearers, raising the concern that the infection is a causative event in colon cancer [78]. In further support, a higher prevalence of colibactin-producing *E. Coli* in the population correlates with a higher incidence of colon and esophageal cancer [79].

### Mutational patterns can track the tissue-of-origin of a cell

Cells from different tissues accumulate somatic mutations at different pace [7, 54, 80, 81]. Differential mutagen exposure is one component determining both the tissue-specific rate of mutation accumulation and the presence of distinct mutational signatures. By exploiting the tissue-specificity of some mutational signatures, researchers have grasped details on the permanence of circulating cells in specific tissues during a lifetime.

UV light, as mentioned before, cannot penetrate the skin, therefore is expected to leave a mutational signature exclusively in skin cells. Surprisingly, the UV-related signature SBS7a represented 10% of mutations in memory T cells from a large study analyzing circulating B and T cells [31]. Memory T cells are a bone-marrow derived cell type, which is known to circulate in the body. Thus, the UV-induced mutational process was used to demonstrate the residency of these cells in the skin, an event that was suspected, but difficult to quantify with existing methodologies. The same was deducted for SBS17, usually observed in gastric and esophageal cancers but here seen in memory cells, indicating exposure to the gastrointestinal mucosa environment [31].

The method can also be applied to other types of cells able to migrate around the body, such as metastases. This use of mutational signatures as markers of tissue-of-origin or tissue-of-residency is attractive for several applications in oncology [82]. Mutational signatures can be used to identify the location of a primary tumor of metastases of unknown origin, outperforming the classification by a trained pathologist [83]. In a 3,668 solid cancer cohort, metastatic tumors derived from colorectal primary cancers displayed an enrichment of the colibactin signature induced by *E. coli* infection. Interestingly, the signature was also observed in metastatic tumors derived from other sites known to be infected by *E.coli* (neck, head, and urinary tract) [77].The identification of tobacco signature in a brain metastasis sample led to the discovery of an undetected primary in the lung of a patient. This case was part of the 100,000 Genomes Project in the UK and highlights the direct clinical benefits of including whole genome sequencing in clinical practice analyses of cancer samples [84].

Even when the organ is known, discerning the specific cancer subtype of a tumor sample can be difficult. Cancer subtypes are most often determined by the origin of the tumor from distinct cell types within the same tissue. Mutational pattern analyses could classify the subtype of a tumor more accurately than standard histological examination [85]. In this case, the primary factor influencing the relationship between the cell-of-origin and the pattern of somatic mutation is not mutagen exposure, which is likely the same across different cell types residing within the same tissue. Instead, it is the epigenetic organization of the genome that plays a crucial role. In fact, chromatin state determines gene expression and is a robust determinant of cell type. But at the same time, chromatin organization affects DNA accessibility to mutagens and DNA repair, thus influencing mutation accumulation [86, 87]. In the brain, cell-type-specific mutation accumulation was shown to be tightly related to the epigenetic organization [88]. In fact, neurons and oligodendrocytes coexist in the same tissue, but present different mutational patterns that correlate with cell-type specific epigenetic marks, chromatin accessibility, and transcriptional levels [88]. Post-mitotic cells, like neurons, were initially thought to have a more stable genome. Instead, they exhibit remarkable somatic mutation rates during adult age [89, 90]. Different studies have characterized the neuron-specific patterns of somatic mutation [88, 91–93], overall strengthening the idea that interphase regulation and architecture of the genome are strong determinants of somatic mutation in aging cells.

### Mutational patterns reflect the activity of the genome maintenance system

The lack of components of DNA repair is a cause of abnormal accumulation of mutations in somatic cells (Fig. 2C). Genetic defects affecting the activity of the homologous recombination and mismatch repair pathways are well-studied examples [82, 94]. The analysis of mutational patterns is becoming an experimental tool to gain a detailed understanding of the molecular activity of DNA-repair enzymes and complexes [95]. Specific genes can be knocked-out in vitro, and the pattern of somatic mutations can be used as a precise output signal to reconstruct the activity of the knocked-out DNA repair component [96–98]. Compared to other functional readouts of enzymatic activity, somatic mutations are very convenient. First, mutations can be detected with exceptional accuracy using the proper technology [99]. Second, mutation signals are easy to quantitatively define, as they are based on the 4 bases code of the genome sequence. Exploiting these concepts, in vitro studies have determined that the knock-out of single DNA-repair genes can lead to unique mutational patterns that describe the specific activity of the depleted protein. Conversely, some DNA-repair components may be considered dispensable or redundant, as their depletion does not induce neither an excessive accumulation of mutations nor a specific mutational signature [97, 98]. There are examples showing that the dependency of DNA repair on specific pathways or pathway components may vary according to cell type and experimental culture conditions (reviewed in [95]). Therefore, a robust assessment of the activity of DNA repair components may be better accomplished by concomitantly testing the effect of a knock-out in multiple cell types or model systems [95].

Familial cancer predisposition syndromes are often caused by inherited loss-of-function mutations in DNA repair components [100]. Patients that inherit defects in DNA repair are expected to show altered accumulation of mutations in their somatic cells, some of which will transform into cancers. Pioneering studies have started to concomitantly analyze mutation landscapes in cancer and normal genomes from these patients [101–104]. This approach offers a precious window on the transition from a normal cell to a cancer, while avoiding the confounding factors of heterogeneous genetic backgrounds and variable environmental exposures. A significant question that can be answered is why the same genome maintenance defect induces cancer-predisposition in some tissues and not in others.

Sporadic cancers are another type of sample that can be used as natural experiments. In this case, the wealth of publicly available data and the heterogeneity of samples can be exploited to uncover unanticipated interactions among DNA repair genes and pathways, and even new mechanisms that influence genome maintenance. Tumor whole exome and genome sequencing data can be used to identify genomes that present pathogenic variants affecting DNA repair genes and to concomitantly evaluate the mutational output of these genetic defects. A systematic analysis in a cohort of 15000 tumor-normal pairs from different tissues has identified many associations between pathogenic germline variants and deviations from the regular somatic mutational pattern [105]. The work examined mutational signatures based on single base and double base substitutions (alterations of 2 consecutive bases), small insertions and deletions, and copy number alterations (larger insertions and deletions). Additionally, researchers explored the distribution of mutations across the genome.

By analyzing all these different mutational features at once, the authors showed that perturbation of the activity of certain DNA-repair pathways generates composite mutational outputs, defined as “mutational phenotypes”. Each mutational phenotype was characterized by various degrees and types of single base substitutions, insertions and deletions, structural variants and alterations of mutation distribution [105]. Another finding is that mutational phenotypes were identified in tumors that did not harbor mutations in known DNA-repair components. In these cases, new genes that influence specific DNA-repair pathways could be inferred from the specific mutational phenotype [105]. One interesting example is mTOR, which is a known cancer driver, but not obviously involved in DNA repair. In particular, mTOR signaling is a key cellular hub connecting the sensing of extracellular stress signals with intracellular adaptation decisions [106]. The analysis showed that mutations in the *MTOR* gene induced a mutational phenotype that resembles loss of mismatch repair, pointing to a quite specific role of mTOR signaling in genome maintenance [105]. More broadly, these findings show that regulation of the genome maintenance system can be an unsuspected part of the oncogenic activity of cancer-related genes, and this feature may happen more often than anticipated.

Along these lines, the loss of cell cycle-related genes *TP53* and *RB* perturbed somatic mutation accumulation, leaving specific mutational signatures [11]. Lack of the p53 (tumor protein 53) and RB (retinoblastoma) proteins promotes tumorigenesis by altering the cell cycle and removing cell cycle arrest signals. There is strong rationale that a dysregulated cell cycle might result in mutation accumulation [107], but studies have so far failed to find a specific mutational signature connected with cell cycle alterations. Starting from an unbiased analysis of mutation distribution along the genome sequence of thousands of cancer genomes, the authors identified mutational signatures that are not characterized by specific nucleotide changes, but by altered mutation distribution, e.g. enrichment of mutations in genomic regions that are usually protected from mutation. Notably, one signature was specifically associated with loss-of-function mutations in *RB* and its pathway, while a distinct signature was associated with loss of *TP53* and its interactors. The study shows that cells that acquire defects in these pathways undergo specific re-organizations of the chromatin, which in turn affect mutation accumulation. Interestingly, these mechanisms occurred similarly across different cancer types, underlining a general mechanism of tumor progression consequent to the loss of two commonly mutated tumor suppressors [11].

In summary, analyses that concomitantly tackle the presence of driver variants and connected somatic mutation patterns can deepen our understanding of how the genome maintenance system works at steady state and after perturbation and are particularly important to clarify the chain of events that convert a normal cell into a cancer. Moreover, such analyses are an exceptionally clever method to mine meaningful information directly from clinical samples and detail the yet very obscure first steps of cancer evolution.

## Conclusions

Here, we have presented examples of how the study of somatic mutations extends beyond their classical use for identification of cancer driver genes in tumor samples. Somatic variants occur at whole genome level, in every cell of the body and throughout the entire life. Detecting somatic mutations across a wide range of tissues and conditions may offer new perspectives on classically challenging aspects of biology, such as tissue changes happening during development, aging and cancer.

## Data Availability

Not applicable.
